# Incidence and excess mortality of hip fracture in young adults: a nationwide population-based cohort study

**DOI:** 10.1186/s12891-016-1166-9

**Published:** 2016-08-05

**Authors:** Tsai-Hsueh Leu, Wei-Chun Chang, Jeff Chien-Fu Lin, Chi Lo, Wen-Miin Liang, Yu-Jun Chang, Dann-Pyng Shih, Cheng-Chun Wu, Chi-Fung Cheng, Sy-Jye Wei

**Affiliations:** 1 Department of Orthopedic Surgery, Wan Fang Hospital, Taipei Medical University, Taipei, Taiwan; 2 Department of Statistics, National Taipei University, Taipei, Taiwan; 3 Department of Hospitality Management, Chung Hua University, Hsinchu, Taiwan; 4 Graduate Institute of Biostatistics, China Medical University, Taichung, Taiwan; 5 Department of Public Health, China Medical University, Taichung, Taiwan; 6 Epidemiology and Biostatistics Center, Changhua Christian Hospital, Changhua, Taiwan; 7 Healthcare System Operation Center, Changhua Christian Hospital, Changhua, Taiwan

**Keywords:** Hip fracture, Incidence, Mortality, Standardized mortality ratio, Young adults, Nationwide population-based study

## Abstract

**Background:**

This study assessed the incidence and excess mortality of hip fractures among inpatients aged 20–40 years in a nationwide population database in Taiwan.

**Methods:**

Subjects were selected from Taiwan’s National Health Insurance Research Database for the period 2001–2008 and were followed up until the end of 2010. A total of 4,523 subjects were admitted for the first time with primary diagnosis of hip fracture and treated with operation.

**Results:**

The overall annual incidence, mortality, and standardized mortality ratio (SMR) decreased from 7.68 to 7.23 per 100,000, from 1.37 % to 0.94 %, and from 9.06 to 6.71, respectively, from 2001 to 2008. The 1-year, 2-year, 3-year, 5-year, and 10-year mortality rates were 1.28 %, 2.44 %, 3.54 %, 5.32 %, and 10.50 %, respectively for the whole cohort. The 1-year, 2-year, 3-year, 5-year, and 10-year SMRs were 8.33, 7.59, 7.28, 6.39, and 5.82, respectively, for the whole cohort. Risk factors for overall death were male gender, trochanteric fracture, hemiarthroplasty, and higher Charlson comorbidity index (CCI) scores.

**Conclusions:**

The high SMRs found in the present study suggest that young adults with former hip fracture should be closely followed up to prevent early mortality.

**Electronic supplementary material:**

The online version of this article (doi:10.1186/s12891-016-1166-9) contains supplementary material, which is available to authorized users.

## Background

Hip fractures in young adults are uncommon and often caused by high-energy trauma, whereas hip fractures in elderly adults are more common and are generally sustained in falls [1–11]. Previous studies estimated that less than 10 % of total hip fractures occurred among subjects aged under 50 to 60 years in North America [12, 13]. Studies likewise revealed that the one-year mortality after hip fractures among the elderly population was as high as 20 % to 30 % [7, 10, 12–18]. Several studies have reported mortality rates in hip fracture patients aged under 50 to 65 years old [1, 5, 7, 10, 11, 18]. However, there are relatively few studies on mortality rates after hip fractures among young adults aged under 40 years [1–11]. Studies from individual institutes generally did not use a large sample size and thus it was not possible to precisely assess the incidence of short-term and long-term mortalities after hip fractures. At present, no population studies have explored the incidence of hip fracture and mortality rates in a young Asian population. Therefore, this study assessed the incidence of hip fracture and excess post-hip fracture mortality using a sample of inpatients aged 20 to 40 years from a nationwide population database in Taiwan.

## Methods

### Data source and subjects

The National Health Insurance (NHI) program in Taiwan was launched in 1995. The program provides compulsory universal medical insurance for all Taiwanese residents. The NHI Research Database (NHIRD), which was established in 1997, is a repository of NHI claims data. As of 2010, the coverage rate in Taiwan’s population of more than 23 million was over 99 %. The completeness and accuracy of the NHI database is verified by Taiwan’s Ministry of Health and Welfare (formerly the Department of Health) and the Bureau of NHI. The data sources in the present study were the NHIRD and the National Register of Deaths Database maintained by Taiwan’s Ministry of Health and Welfare.

This study screened all subjects, aged between 20 and 40 years and admitted to hospitals between January 1, 2001 and December 31, 2008. All subjects were followed up to death, exit from the NHI program, or the end of 2010. All subjects were followed up for 2–10 years, depending on when the patients were entered into the study. The following two conditions represented the inclusion criteria for such subjects: (i) the first discharge diagnosis code was hip fracture [based on the International Classification of Disease, Ninth Revision, Clinical Modification (ICD-9-CM) codes 820, 820.0, 820.00, 820.01, 820.02, 820.09, 820.8, 820.03, 820.2, 820.20, and 820.21] and (ii) the medical code was surgery of internal fixation or hemiarthroplasty (based on ICD-9-CM codes 79.15, 79.35, and 81.52). The first admission date of hip fracture was defined as the index date. The exclusion criteria for the inpatients admitted to hospitals were pathological fractures (ICD-9-CM codes 733.14 and 733.15) or open hip fractures (ICD-9-CM codes 820.1, 820.10, 820.11, 820.12, 820.19, 820.9, 820.13, 820.22, 820.3, 820.30, 820.31, and 820.32). Patients who had surgery on the pelvis, femur, or hip regions before the index date were excluded to avoid confounding effects. More than 99 % of the hip fractures in these young adults were caused by high-energy trauma; i.e., most were due to motorbike accident. Multiple fractures and concomitant head and neck fractures were possible but the latter type was relatively rare.

### Outcome measures

This study analyzed several outcomes, namely, (a) annual incidence of hip fracture; (b) annual mortality; (c) annual standardized mortality ratio (SMR); (d) cumulative mortality; (e) follow-up SMR; and (f) risk factors of mortality, over ten years after hip fracture among young adults. Annual mortality and cumulative mortality are absolute rates indicating the occurrence proportion of death among these study subjects. Meanwhile, annual SMR and follow-up SMR are relative mortality rates indicating the relative risk of death of our study population compared to that in the corresponding general population. Overall survival time was defined as the duration from the index day to the death day. Subjects alive at the end of the study or lost to follow-up were treated as censored. The comorbidities of each subject were retrieved before or at the time of the index day based on the Charlson comorbidity index (CCI) [19].

### Statistical analysis

For each cohort year, we calculated the annual incidence as the number of inpatients with hip fractures divided by the mid-population in that cohort year, and stratified patients by gender. We calculated the annual mortality as the number of deaths divided by the number of newly diagnosed cases in that cohort year, and stratified patients by gender. We calculated the annual standardized mortality ratio (SMR) from the date of diagnosis of hip fracture in that calendar year and then followed up the patient for one year. We estimated the overall cumulative mortality based on Kaplan–Meier (KM) method. We calculated the one- to ten-year follow-up SMRs of hip fracture based on the available data after the fracture, and stratified them by age and gender. The SMR estimation was based on the following definition: the number of deaths among inpatients with hip fractures divided by the expected number of death cases according to the age-, gender-, and calendar-year-specific death rates obtained from the National Register of Deaths Database of Taiwan. We assessed the excess mortality in incident hip fracture patients with that of the general population using annual and follow-up SMR. We explored the effects of risk factors, such as age, gender, type of hip fracture, and CCI score, on mortality using the log-rank test. All analyses were performed using the SAS System (version 9.2; SAS Institute, Cary, NC).

## Results

Between 2001 and 2008, 4,523 subjects were admitted for the first time with a primary diagnosis of hip fracture and then treated with surgery. Among these patients, 3439 (76.03 %) were male, 1084 (24.97 %) were female, 1931 (42.69 %) had trochanteric fracture, 2592 (57.31 %) had cervical fracture, 4363 (96.46 %) received internal fixation, and 160 (3.54 %) received hemiarthroplasty (Table 1). For the period 2001 to 2008, the annual incidence rates of hip fractures changed from 7.68 to 7.23 (*P* =0.6519), from 11.09 to 11.29 (*P* =0.9885), and from 4.15 to 3.07 (*P* =0.5415) per 100,000 for the overall population, males, and females, respectively (Table 2).

**Table 1:** Baseline characteristics of hip fracture among young adults in Taiwan

		Total *N =* 4,523	Male *N =* 3,439	Female *N =* 1,084	*P* ^*a*^
Age, mean ± SD (years)		31.03 ± 5.93	31.19 ± 5.85	30.53 ± 6.17	<0.0001
Year, N (%)	2001	585 (12.93)	430 (12.50)	155 (14.30)	0.0344
	2002	553 (12.23)	415 (12.07)	138 (12.73)	
	2003	551 (12.18)	433 (12.59)	118 (10.89)	
	2004	589 (13.02)	456 (13.26)	133 (12.27)	
	2005	611 (13.51)	462 (13.43)	149 (13.75)	
	2006	563 (12.45)	438 (12.74)	125 (11.53)	
	2007	537 (11.87)	383 (11.14)	154 (14.21)	
	2008	534 (11.81)	422 (12.27)	112 (10.33)	
Hip fracture, N (%)	Trochanteric	1931 (42.69)	1580 (45.94)	351 (32.38)	<0.0001
	Cervical	2592 (57.31)	1859 (54.06)	733 (67.62)	
Operation, N (%)	Internal fixation	4363 (96.46)	3313 (96.34)	1050 (96.86)	0.4125
	Hemiarthroplasty	160 (3.54)	126 (3.66)	34 (3.14)	
Charlson score, N (%)	0	4110 (90.87)	3103 (90.23)	1007 (92.9)	0.0267
	1	267 (5.90)	219 (6.37)	48 (4.43)	
	≥2	146 (3.23)	117 (3.40)	29 (2.68)	
Any comorbidity, N (%)					
	Hypertension	116 (2.56)	102 (2.96)	14 (1.28)	0.0024
	Diabetes mellitus	102 (2.26)	85 (2.48)	17 (1.56)	0.0807
	Heart disease	50 (1.11)	38 (1.11)	12 (1.10)	0.9955
	Chronic pulmonary disease	58 (1.28)	47 (1.37)	11 (1.01)	0.3692
	Chronic liver disease	181 (4.00)	165 (4.81)	15 (1.37)	<.0001
	Chronic renal disease	45 (1.00)	30 (0.88)	15 (1.37)	0.1390
	Cerebrovascular disease	44 (0.98)	34 (1.00)	10 (0.92)	0.0807
	Cancer	102 (2.26)	57 (1.65)	46 (4.22)	<.0001

^a^The *P* value were calculated based on *t* test or Chi-squared test, which was used to assess the gender difference

**Table 2:** Annual incidence, mortality and SMR of hip fracture among young adults in Taiwan

	Incidence (95 % CI)^a^			Mortality (95 % CI) ^b^			SMR(95 % CI)^c^		
	Overall	Male	Female	Overall	Male	Female	Overall	Male	Female
2001	7.68(7.06–8.30)	11.09(10.04–12.13)	4.15(3.49–4.80)	1.37(0.43–2.31)	1.86(0.58–3.14)	0.00(0.00–0.00)	9.06(8.82–9.30)	10.32(10.02–10.62)	0.00(0.00–0.00)
2002	7.25(6.65–7.86)	10.70(9.67–11.73)	3.69(3.07–4.30)	1.45(0.45–2.44)	1.93(0.60–3.25)	0.00(0.00–0.00)	9.82(9.56–10.08)	11.04(10.72–11.36)	0.00(0.00–0.00)
2003	7.24(6.64–7.85)	11.19(10.14–12.24)	3.16(2.59–3.73)	1.81(0.70–2.93)	1.85(0.58–3.12)	1.69(0.00–4.02)	11.42(11.14–11.70)	10.07(9.77–10.37)	24.67(23.77–25.57)
2004	7.77(7.15–8.40)	11.84(10.75–12.92)	3.57(2.96–4.18)	1.19(0.31–2.06)	1.32(0.27–2.36)	0.75(0.00–2.22)	7.27(7.05–7.49)	6.86(6.62–7.10)	11.42(10.85–11.99)
2005	8.10(7.46–8.75)	12.06(10.96–13.16)	4.02(3.37–4.66)	1.31(0.41–2.21)	1.30(0.27–2.33)	1.34(0.00–3.19)	8.39(8.16–8.62)	7.00(6.76–7.24)	20.83(20.10–21.56)
2006	7.52(6.90–8.15)	11.54(10.46–12.62)	3.39(2.80–3.99)	0.89(0.11–1.66)	0.68(0.00–1.46)	1.60(0.00–3.80)	5.73(5.53–5.93)	3.78(3.60–3.96)	25.66(24.77–26.55)
2007	7.23(6.62–7.84)	10.18(9.16–11.20)	4.20(3.54–4.87)	1.12(0.23–2.01)	1.31(0.17–2.44)	0.65(0.00–1.92)	8.19(7.95–8.43)	7.78(7.50–8.06)	11.03(10.51–11.55)
2008	7.23(6.61–7.84)	11.29(10.21–12.37)	3.07(2.50–3.63)	0.94(0.12–1.75)	0.95(0.02–1.87)	0.89(0.00–2.64)	6.71(6.49–6.93)	5.89(5.66–6.12)	15.04(14.32–15.76)
*P* ^d^	0.6519	0.9885	0.5415	0.0464	0.0094	0.2806	0.0781	0.0241	0.2043

^a^Incidence = the number of new cases/the population total in the middle of the calendar year * 100,000

^b^Mortality = the number of deaths/the number of new cases in the calendar year * 100

^c^Annual SMRs were calculated from the date of first diagnosis of hip fracture in that calendar year to one year later

^d^The *P* value were calculated based on *t* test, which was used to assess the trend of the rates/ratios increased/decreased with the calender years

For the same period, the annual mortality rates of hip fractures decreased significantly from 1.37 % to 0.94 % (*P* =0.0464) and from 1.86 % to 0.95 % (*P* =0.0094) for the overall population and males, respectively (Table 2). The values in females were unstable due to the small number of newly diagnosed hip fracture cases which resulted in fewer death cases within one year after fracture. We used the annual SMR to indirectly compare the annual mortality of young adults after hip fracture with that of the general population in Taiwan. The overall annual SMR was 9.06 in 2001 which decreased to 6.71 in 2008 (*P* =0.0781) (Table 2).

**Fig. 1:**
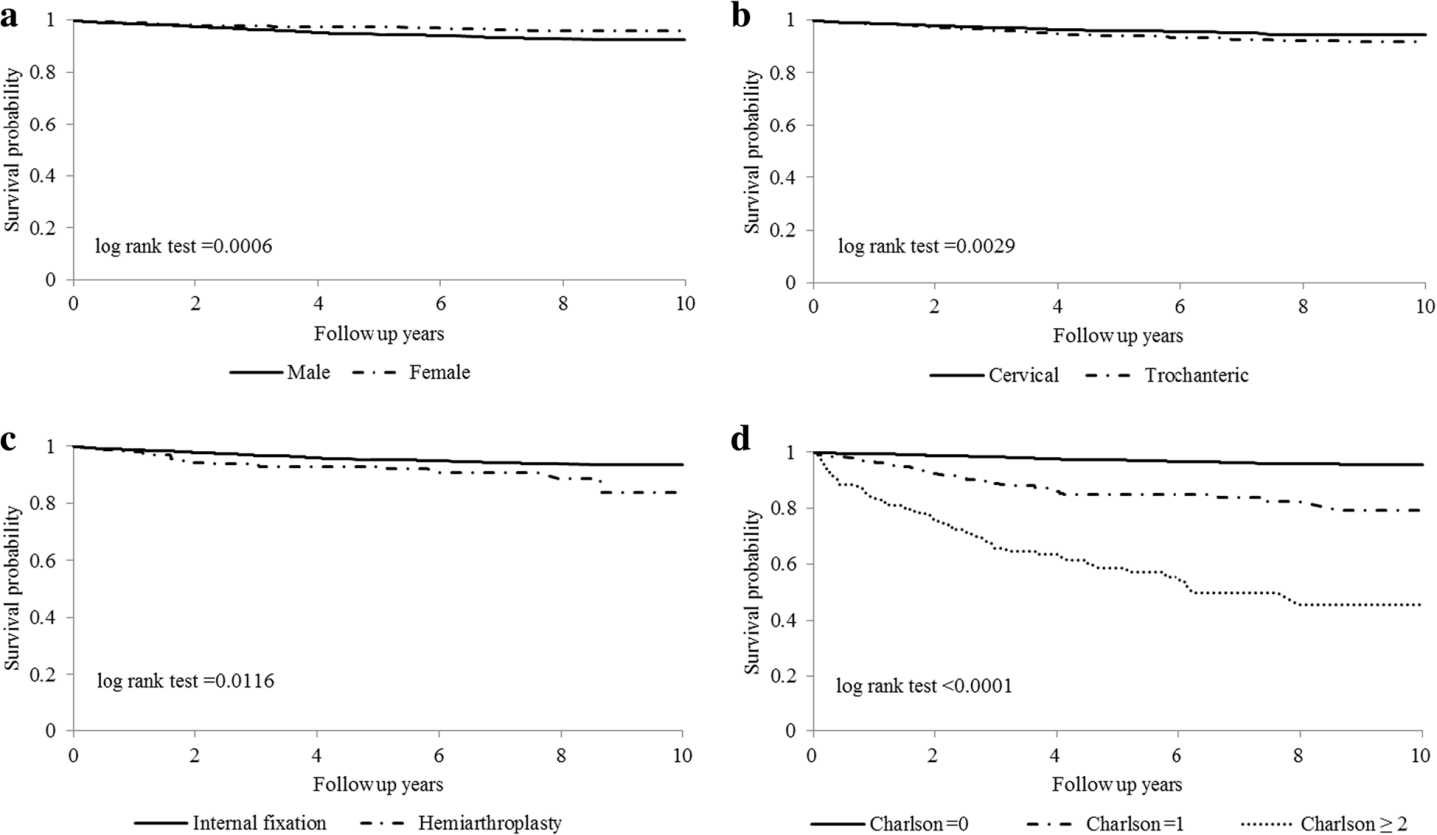
Ten-year overall survival curves stratified by (**a**) gender, (**b**) fracture type, (**c**) operation type, and (**d**) Charlson comorbidity index score

For the entire cohort, the 1-year, 2-year, 3-year, 5-year, and 10-year follow-up mortality rates were 1.28 %, 2.44 %, 3.54 %, 5.32 %, and 10.50 %, respectively (Table 3). Males had higher mortalities than those of females within ten years after the occurrence of fracture (Table 3 and Fig. 1). The gender- and age-stratified follow-up mortalities indicated that all male age groups had higher death rates than those of females within ten years after the occurrence of fracture, except the 20–24 years group (Table 3). For the entire cohort, the 1-year, 2-year, 3-year, 5-year, and 10-year SMRs were 8.33, 7.59, 7.28, 6.39, and 5.82, respectively. Females had higher SMRs than those of males within ten years after the occurrence of fracture (Table 3). The gender- and age-stratified SMRs indicated that all female age groups had higher SMRs than those of males within ten years after the occurrence of fracture (Table 3). We compared the effects of the possible risk factors of mortality using a log-rank test (Fig. 1). The statistically significant risk factors for overall death were male gender, trochanteric fracture, hemiarthroplasty, and larger CCI scores.

**Table 3:** One- to ten-year follow-up mortality rates and SMRs of hip fracture stratified by gender and age among young adults in Taiwan

			Mortality(%)^b^					SMR				
		N^a^	1-year	2-year	3-year	5-year	10-year	1-year	2-year	3-year	5-year	10-year
Total												
	Total	4,523	1.28	2.44	3.54	5.32	10.50	8.33	7.59	7.28	6.39	5.82
	Male	3,439	1.45	2.69	3.98	6.13	12.02	7.82	6.91	6.83	6.13	5.54
	Female	1,084	0.74	1.65	2.13	2.69	5.32	12.90	13.57	11.24	8.66	8.20
20–24 years												
	Total	961	0.21	0.62	1.05	1.63	2.32	2.75	4.17	4.30	3.49	3.42
	Male	687	0.14	0.58	1.03	1.83	2.09	0.00	2.46	2.81	2.61	2.29
	Female	274	0.36	0.73	1.09	1.09	2.96	18.05	13.69	12.67	8.52	9.95
25–29 years												
	Total	959	0.93	1.24	1.93	3.51	9.27	7.66	5.27	5.60	6.09	5.26
	Male	723	1.10	1.24	2.14	3.89	10.27	7.53	4.31	5.19	5.62	4.95
	Female	236	0.42	1.26	1.26	2.33	5.96	8.68	13.00	8.93	9.89	7.79
30–34 years												
	Total	1,075	1.48	2.96	4.05	5.58	11.86	10.15	9.54	8.33	6.94	6.35
	Male	846	1.53	3.29	4.41	5.93	13.05	9.17	9.16	8.02	6.58	5.98
	Female	229	1.31	1.75	2.67	4.30	4.30	20.32	13.50	11.62	10.73	10.16
35–39 years												
	Total	1,528	2.02	3.97	5.76	8.56	15.53	8.84	8.07	7.90	6.80	6.22
	Male	1,183	2.35	4.37	6.51	10.13	18.29	8.80	7.46	7.53	6.74	6.13
	Female	345	0.86	2.59	3.18	3.18	6.17	9.26	13.77	11.42	7.39	6.94

^a^The N represents the number of young adults with hip fracture enrollerd initially in the study

^b^The follow-up mortality rates were estimated using Kaplan–Meier (KM) method

## Discussion

The present investigation is the first population study to evaluate long-term excess mortality in young adults after hip fracture in Taiwan. The annual mortality and SMR of young adults after hip fracture increased gradually during 2001–2003 and decreased slightly after 2005. The launch of the national health insurance program in 1995 and the nationwide implementation of a case payment system in 2002–2003 provided better funding for hip fracture patients and allowed for the provision of more complete care. The decreasing trend of annual SMR may indicate that following the implementation of the NHI there was a decrease in mortality rates for young adults in the general population and also a decrease in mortality rates for young adults with hip fracture, but there was higher decreases in the mortality rate of young adult hip fracture patients compared with that of the general population of young adults. The general improvement in healthcare, as well as the year-on-year improvement in surgical techniques, the increased penality for drunk driving, and decreasing traffic accident death rates may explain, at least in part, the decrease in peri-operative mortality and short-term post-operative mortality after 2003.

The estimated 1-month, 3-month, 6-month, 1-year, 2-year, 3-year, 5-year, and 10-year follow-up mortality rates were 0.13 %, 0.48 %, 0.81 %, 1.28 %, 2.44 %, 3.54 %, 5.32 %, and 10.50 %, respectively (Table 3). Karantana et al. revealed that the cumulative mortality rates were 3.5 % and 13.3 % for 30 days and 1 year after hip fracture among women under 65 years [10]. The differences observed might be attributed to the selection of a greater number of subjects aged over 40 years in the Karantana et al. report [10]. The overall follow-up 1-year, 2-year, 5-year, and 10-year SMRs were 8.33, 7.59, 7.28, 6.39, and 5.82, respectively. Several studies demonstrated that hip fractures continuously affected the long-term mortality even one year after the occurrence of fracture among elderly adults [20–22]. Our results support the premise that hip fractures affected both short-term and long-term mortality rates. To our best knowledge, the present investigation is the first study of long-term excess mortality among young adults. Furthermore, we analyzed the causes of death stratified by year of death for up to ten years following the index day (Additional file 1: Table S1). We found that accident injury, cancer, and suicide were the major causes of death, each of which is highly correlated with the mortality rate of the young adult population. Though the proportion of each of these causes of death fluctuated slightly from year to year, overall the contribution of each major cause to the death rate remained stable.

We also determined that the long-term mortalities and SMRs varied by gender and age in Taiwan, as indicated in the literature on elderly adults [23–25]. We found that in the long-term mortalities stratified by gender and age, the young male group had a higher mortality than that of the young female group, while the result for SMRs was reversed within ten years after the occurrence of fracture (Table 3). One of the reasons for the higher risk of mortality in males might involve the greater prevalence of high-energy trauma with severe injury in males than in females, which might result from differences in behaviors such as vehicular speeding and lifestyle factors between the genders. Meanwhile, high-energy trauma causes the most hip fractures among young adults, and females might be less biomechanically resilient than male, resulting in worse outcomes following a traumatic impact for females when compared to those in their corresponding gender-specific subgroup in the general population. With increasing age, hip fractures tend to show a greater correlation with osteoporosis or osteopenia. Several studies reported that the prevalence of osteoporosis rapidly increased after the age of 40 to 45 years [26–29]. Other studies revealed that the incidence of osteoporotic hip fractures increased after 40 to 50 years of age [10, 28, 30, 31]. We postulate that the prevalence of osteopenia begins to increase even before the age of 40, and contributes to the increased mortality after hip fractures.

We found that the annual incidences of hip fracture among young adults decreased from 7.68 to 7.23, 10.18 to 11.84, and 3.07 to 4.20 per 100,000 persons per year for overall, males, and females, respectively, from 1999 to 2008. These incidence rates of hip fractures among young adults were lower than those of elderly adults [18, 30, 32–34]. Wang et al. reported that the incidence rates of hip fractures among elderly adults were 405 and 476 per 100,000 persons in 1998 and 2009, respectively [34]. Only a few studies have reported population incidence rates of hip fractures among young adults [5, 7, 18, 30, 32, 35]. Previous studies reported that the annual incidence rates of hip fracture varied from 1.9 to 16.3 per 100,000 persons per year [5, 7, 18, 30, 32, 35]. These incidence rates of hip fractures among young adults were close to those reported in Germany [36] and Japan [30], but higher than those in England [18] and Spain [35]. The incidence rates of hip fractures among young adults fluctuated in Taiwan, showing a similar pattern to that observed in England [18]. However, Icks et al. revealed that the incidence rates of hip fractures gradually decreased from 1995 to 2004 [32]. Possible factors that contributed to the differences among regions include different inclusion criteria, distributions of age and gender in the studied populations, trauma severity, and bone mineral density near middle age.

The statistically significant risk factors of overall death were male gender, older age, trochanteric fracture, hemiarthroplasty, and larger CCI scores. These risk factors for mortality after hip fractures among young adults in Taiwan were similar to those reported for the elderly population [10, 17, 34, 37]. Only a few studies analyzed the risk factors, including comorbidities and lifestyles, for the mortality among young adults [10, 11]. We found that among young adults, males had a higher risk for overall death than females. Moreover, males had a higher prevalence of multiple comorbidities, especially chronic pulmonary disease, than females, which might explain one of the reasons for the higher risk of mortality. Duckworth et al. revealed that excess alcohol consumption and pre-existing morbidities such as renal, liver, and respiratory diseases, were predictors of surgery failure among 122 adult fracture patients younger than 60 years [11]. Karantana et al. investigated 315 hip fractures among younger women aged less than 65 years, and determined that smoking and alcohol abuse were important risk factors for mortality [10]. In their meta-analysis, Hu et al. identified multiple comorbidities as risk factors of overall mortality among elderly adults [37]. However, the comorbidities used in the multivariable analysis varied among these studies. There were no clinical measurements of individuals in our database that could be used to sufficiently evaluate these risk factors. We used CCI score to represent the combined severity of multiple comorbidities, which was demonstrated to play a key role in the mortality rate after hip fractures [38, 39]. Hip fractures among young adults are typically treated with internal fixation. We found that hemiarthroplasty had a higher mortality rate compared with that of internal fixation. However, only 160 subjects (3.54 %) received hemiarthroplasty in our database; the sample size was thus much too small to obtain a meaningful conclusion of the effect of surgery type.

### Limitations

Our study used a sample of young adults in Taiwan, particularly hospitalized patients who had hip fractures and subsequently underwent surgery. Selection biases may have existed and thus, our results should be interpreted with caution. Our database had incomplete clinical data for this population and therefore there may have been a number of potentially influential variables that changed or were not taken into consideration during the follow-up period, such as pre-operative joint functions/conditions, smoking status, body mass index, bone mineral density, lifestyle, severity of comorbidity, and quality of life. Furthermore, in contrast to other case–control and cohort studies, there was no control group in the present study. We did not directly compare the relative risk of death to that of a population without hip fractures or to a population with hip fractures but without surgery. We used the NHI database of inpatients in this study, which includes all subjects admitted to hospitals in Taiwan. Since many young adults were admitted to hospitals due to severe medical diseases, surgical diseases or major trauma, it was very difficult to find a well-defined matched control group that did not have a hip fracture nor any other major disease to achieve a balanced distribution in covariates/comorbidities between the two groups, with or without hip fracture. Instead, we calculated the SMRs from Taiwan’s National Register of Deaths Database to evaluate indirectly the relative risk of death compared to that of the general population.

## Conclusions

The overall annual incidence rates of hip fracture for young adults aged 20 to 40 years in Taiwan were 7.68 and 7.23 per 100,000 in 2001 and 2008, respectively. The cumulative mortality rate was as high as 10.5 % during the ten-year follow-up. Although the one- to ten-year follow-up SMRs decreased, all values remained five-fold greater or more, which indicated that the relative impact on the mortality rate was quite high in young adults with hip fracture compared with the mortality rate in the general young adult population. We recommend that young adults that have been treated for hip fracture should be closely followed up to reduce the risk of mortality.

## Abbreviations

CCI, Charlson comorbidity index; ICD-9-CM, international classification of disease, ninth revision, clinical modification; IRB, institutional review board; NHI, The National Health Insurance; NHIRD, The NHI Research Database; SMR, standardized mortality ratio

## Additional file

Additional file 1: Table S1. Proportions of leading causes of death stratified by year of death after hip fracture. (DOC 58 kb)
