# Supplementary material for: Incidence and excess mortality of hip fracture in young adults: a nationwide population-based cohort study
Source: BMC Musculoskelet Disord. 2016 Aug 5;17:326. doi: 10.1186/s12891-016-1166-9 (PMC4974740; doi:10.1186/s12891-016-1166-9)
Supplement: Additional file 1: Table S1. — Proportions of leading causes of death stratified by year of death after hip fracture. (DOC 58 kb) [file 12891_2016_1166_MOESM1_ESM.doc]

**Table S1. Proportions of leading causes of death stratified by year of death after hip fracture**

|  | Causes of Death (%) | | | | | | | | | |
| --- | --- | --- | --- | --- | --- | --- | --- | --- | --- | --- |
| Causes | 1-year | 2-year | 3-year | 4-year | 5-year | 6-year | 7-year | 8-year | 9-year | 10-year |
| Accidental injury | 32.50 | 29.09 | 27.07 | 26.20 | 27.00 | 25.29 | 24.58 | 23.27 | 22.09 | 23.52 |
| Malignancy | 17.47 | 17.86 | 18.47 | 17.66 | 18.92 | 17.29 | 16.23 | 17.67 | 18.33 | 18.24 |
| Suicide | 8.44 | 9.74 | 11.73 | 12.48 | 14.29 | 16.86 | 17.96 | 16.58 | 17.95 | 17.80 |
| Chronic liver disease and cirrhosis | 6.99 | 6.90 | 5.48 | 6.13 | 6.33 | 6.50 | 6.06 | 6.89 | 5.92 | 6.12 |
| Heart disease | 3.75 | 3.92 | 3.73 | 4.05 | 4.23 | 4.42 | 4.41 | 5.30 | 5.62 | 5.29 |
| Cerebrovascular diseases | 2.95 | 3.24 | 2.68 | 2.86 | 3.12 | 3.35 | 2.65 | 2.90 | 2.77 | 2.57 |
| Diabetes | 1.12 | 1.27 | 1.39 | 1.63 | 1.15 | 1.54 | 1.39 | 1.56 | 1.26 | 1.12 |
| Nephritis, nephrotic syndrome, and nephrosis | 0.88 | 0.90 | 0.79 | 0.76 | 0.79 | 0.63 | 0.72 | 0.79 | 0.70 | 0.75 |
| Pneumonia | 0.70 | 0.65 | 0.85 | 1.34 | 1.15 | 1.08 | 1.08 | 0.88 | 1.39 | 1.19 |
| Tuberculosis | 0.63 | 0.64 | 0.29 | 0.51 | 0.25 | 0.23 | 0.20 | 0.25 | 0.22 | 0.33 |
| Bronchitis, emphysema, and asthma | 0.29 | 0.53 | 0.47 | 0.43 | 0.39 | 0.48 | 0.53 | 0.52 | 0.53 | 0.55 |
| Septicemia | 0.23 | 0.36 | 0.27 | 0.29 | 0.28 | 0.21 | 0.25 | 0.22 | 1.02 | 0.98 |
| Ulcers of the stomach and duodenum | 0.20 | 0.15 | 0.16 | 0.20 | 0.13 | 0.19 | 0.12 | 0.17 | 0.07 | 0.07 |
| Hypertensive disease | 0.15 | 0.13 | 0.11 | 0.14 | 0.13 | 0.11 | 0.22 | 0.18 | 0.38 | 0.53 |
